# Learning from mistakes climate scale: Development and validation

**DOI:** 10.3389/fpsyg.2022.911311

**Published:** 2022-08-08

**Authors:** Michelle Chin Chin Lee, Su Woan Wo

**Affiliations:** ^1^School of Psychology, Massey University, Auckland, New Zealand; ^2^Department of Psychology, Sunway University, Bandar Sunway, Malaysia

**Keywords:** scale development, validation, Malaysia, employee, learning from mistakes climate

## Abstract

Learning from mistakes plays an important role in employee development; however, such a learning scale has not yet been developed. The objective of this study was to develop and examine the psychometric properties of the Learning from Mistakes Climate Scale (LMCS) in Malaysia. A pool of items was first developed based on the literature, with an expert panel then convened to select items that met the definition of learning from mistake climate in the workplace, specifically in Malaysia. The experts agreed on 23 items to be rated. In total, 554 working adults with a mean age of 32.28 were then recruited for this study. The LMCS was administered at baseline and 10–14 days later as a retest: 468 participants took part in the retest study, a dropout rate of 15.52%. Confirmatory factor analysis showed that the LMCS is a 17-item one-factor model. Validity, in its various forms, was supported, namely convergent validity, criterion validity, and predictive validity. Analysis also showed significant reliability, that is, test–retest reliability and in all intra-class correlations. The LMCS was found to be a valid and reliable instrument to assess the learning from mistake climate in Malaysia. This is the first scale in the organizational learning climate literature to integrate the mistake tolerance aspect. This instrument can assist in creating a psychologically safe work environment that helps to facilitate learning, especially in a highly hierarchical, collectivistic culture that is high in power distance.

## Introduction

Work dynamics are constantly changing in today’s world: to effectively and efficiently capture market opportunities, organizations are continually updating knowledge and acquiring new information to satisfy the needs of their customers (Fraj et al., 2015; Kasim, 2015; Jogaratnam, 2017). To maintain these high work demands, employees are often working around the clock to ensure successful delivery of projects, with their work indirectly translating into higher job performance and higher work productivity. In addition, employees are also constantly upgrading their knowledge and skills to adapt to organizational demands (Abd Awang et al., 2010; Hanaysha, 2016). In adaptation to and fulfillment of global and organizational demands, mistakes are bound to happen in the process of learning and skill acquisition (Baumard and Starbuck, 2005). In most organizations, making mistakes is frowned upon and is something for which employees may be punished (Weinzimmer and Esken, 2017). As a result, mistakes are often covered up, hidden, and/or remain unreported, with employees hoping that the matter will resolve itself. However, such behaviors invite future mistakes and prevent the sharing of information about these mistakes, resulting in other employees not being able to learn from them to improve their work performance (Wimer and Nowack, 1998).

The concept of learning from mistakes has remained a challenge for both research and business practice (Harteis and Buschmeyer, 2012) for a few reasons. Firstly, organizations do not like to associate with mistakes and failures, instead focusing only on successes to avoid damaging their reputation (Mittelstaedt, 2004). As a result, the literature lacks breadth and depth on this topic. The most recent studies on learning from mistakes were conducted more than 15 years ago (Tucker and Edmondson, 2003; Edmondson, 2004). These studies revealed that learning from mistakes has a positive impact on employees. Over nearly two decades, few studies have been conducted on mistakes and learning; therefore, it is evident that the literature has not placed sufficient emphasis on this aspect in the learning literature. This identifies the need to re-emphasize the creation of a more mistake-tolerant organization in the employee learning process, and even more so within the Asian setting which is highly hierarchical and collectivistic (Lee et al., 2017). With these dynamics, mistakes are frowned upon, with this encouraging employees to hide mistakes to avoid reprimands or punishment from management or the organization (Pelletier, 2010).

Secondly, few research findings are available on the relationship between learning from mistakes and work-related issues. Most of the literature on learning focuses on the learning aspect which does not entail investigation of the type of environment that allows learning to thrive (Mikkelsen and Grønhaug, 1999). It is only with the more recent development of a learning climate scale by Nikolova et al. (2014) that the literature has started to emphasize the provision of resources and appreciation by the organization to facilitate the learning process. Thus, the current study acknowledges the organizational context that is needed to allow learning to happen. The study argues that making mistakes is an important aspect for learning to occur. These gaps in the literature warrant an important review of what constitutes learning from mistakes and how it can be viewed from a larger picture (i.e., the organizational perspective; Tucker and Edmondson, 2003). Whether employees are encouraged to make mistakes is often ingrained in the system established within the organization.

Finally, most studies have investigated learning from mistakes at the individual level (Weinzimmer and Esken, 2017). The danger of viewing mistakes from this level is that it disregards the organizational aspect which conveys the overall climate regarding making mistakes (Hunt and Ivergard, 2007). The expected values and behaviors expected of employees are usually conveyed by the overall climate, hence influencing employees’ behavior (Schneider et al., 2013). In addition, measurement at the individual level often results in subjective evaluations that are biased (Wetzel et al., 2016). This does not allow the mistake learning literature to conduct a fair investigation which should be viewed through an organizational lens rather than through an individual lens, following the approach by Nikolova et al. (2014).

The current study therefore proposes to develop and validate a newly established scale to see how it relates to first-order and second-order problem-solving behavior. In this way, it is intended to develop a better understanding of the nature, meaning, and impact of mistake learning and work relationships. This study also seeks to understand the intricate relationship between employees and leaders from an interpersonal perspective. Hence, appropriate coaching and direction setting from leaders, co-workers, and human resources management can provide guidance to deal with future incidents, in this way ensuring a conducive working environment that is safe, open, and receptive to mistakes. However, despite a few studies having been conducted on mistake learning, research on this topic to date remains scarce in the literature (Carmeli and Gittell, 2009).

This study aims to assist in the creation of a psychologically safe working environment in which optimal learning can occur, especially in a country that is hierarchical, collectivistic, and high in power distance. Developing and utilizing a scale that measures organization-level mistake learning will allow human resources personnel to measure, review, and adopt more employee-friendly policies that facilitate learning (Probst et al., 2019). This would allow learning to be maximized not only through formal training, but also when carrying out their daily work tasks. Human resources personnel would then be able to train managers at all levels to ensure they worked in alignment with the established policies (Beer, 1997). This would indirectly improve the leader–employee relationship, creating a better understanding about the consequences of mistakes and ways to overcome them. This study is considered a precedent for future research on employee and organizational learning that focuses on making and learning from mistakes.

## Review of the literature and hypotheses development

### Learning climate and learning from mistake climate

Learning is defined as “changes in the behavior of an organism that result from regularities in the environment of the organism” (De Houwer et al., 2013). Learning is part of an individual’s life and has an important place within the organization. As the world rapidly develops, many changes are happening to which the organization must adapt, even aiming to be at the forefront to have a competitive edge within that industry (Liao and Wu, 2010). Employees must have the most up-to-date skills to cope with the demands of today and tomorrow. Learning plays an important role in the employee development process; hence, organizations have introduced various types of training and workshops so employees will have the latest skills (Ibrahim et al., 2017).

The learning climate concept is defined as perceptions of work settings that help or hinder learning at work (Nixon, 1991). It is also defined as the organization’s beneficial activities that help employees to create, acquire, and transfer knowledge (Eldor and Harpaz, 2016). Its focus is on the overall environment of learning opportunities provided by the organization. In another study, a high learning climate was found to be important for higher motivation for transfer learning and lower turnover intention (Edmondson, 2004). This shows the importance of having a high learning climate within the organization to improve the organization’s overall productivity and performance (Maruping and Magni, 2012).

Organizational climate has become a new focus area within the industrial/organizational literature, with this construct viewed from an organizational perspective or a team perspective, and not from an individual perspective (Choudhury, 2011). Studies in the literature have also commented on the danger of relying on individual responses that can be biased and subjective. The use of the organizational climate construct is also important as it acknowledges the effect of the environment factor on employees’ behaviors and work outcomes.

The learning process is usually carried out in a formal and organized manner through workshops (Jehanzeb and Bashir, 2013). However, learning can also happen when doing daily tasks and, during these times, mistakes are bound to happen (Harteis et al., 2008). While one meta-analysis study has shown that informal learning also assists in knowledge/skill acquisition and work performance (Cerasoli et al., 2018), few studies have been conducted on this topic. The literature on the overall learning process climate is lacking. More specifically, a natural response is triggered in employees when mistakes occur, and they need to deal with the mistakes and their consequences. Employees in punitive organizations usually do not dare to report such incidents to avoid being reprimanded (Weinzimmer and Esken, 2017).

On the other hand, employees in organizations that embrace mistakes will report and share their problems. It is only when problems are shared that organizations can learn from them (Ganguly et al., 2019). In line with that argument, a learning from mistake climate within the organization plays an important role in how employees view and respond to those mistakes. They can either communicate to other employees for advice and for the purpose of improvement or they can hide those mistakes and pretend they never happened (Ellis and Davidi, 2005). The current study therefore aims to develop and validate a learning from mistakes climate scale. We define mistake learning as “a collective perception of … employees in the tolerance and acceptance level of [the] organization in making mistakes and taking it as a learning process.”

The words “mistake” and “error” are often used interchangeably, but these words have some differences (Hon, 1995). The word “error” is often directed toward the system and actions that did not result in expected outcomes, often meaning unexpected results due to carelessness. These errors do not pose a danger to the organization’s overall outcomes (Bligh et al., 2018). The word “mistake,” on the other hand, refers to decisions made when carrying out a task that result in unexpected outcomes, often incurring a high cost if the mistake is not amended. In addition, error usually refers to the task or system, while mistake usually refers to the individual (Weinzimmer and Esken, 2017). From the locus of control perspective, error is externally focused (i.e., toward the system) while mistake is internally focused (i.e., toward the individual). Based on that argument, a mistake is more related to an individual’s action, outcome, and responsibility.

### Learning from mistakes climate and psychological safety climate

As the learning from mistake climate is defined in our study as the acceptance and tolerance of the organization for employees to make mistakes during normal tasks and to take those mistakes as a learning process, this signifies a working environment that welcomes mistakes and is not punitive in nature (Gu et al., 2013). Hence, it clearly relates to the psychological safety climate. Studies have also found that organizations with a high tolerance toward mistakes have employees with a higher level of learning (Weinzimmer and Esken, 2017).

While these two forms of climate are similar with both relating to creating an environment that focuses on employee mastery (Men et al., 2020), they have their individual distinctiveness. Firstly, the psychological safety climate emphasizes employees’ psychological safety (Casey et al., 2017), while the learning from mistake climate emphasizes learning through mistakes. Secondly, the psychological safety climate is more linked to the voice and justice climate (Walumbwa and Schaubroeck, 2009), while the learning from mistake climate is more linked to the learning climate. Nevertheless, we see close links with these two forms of climate which still retain their distinctive roles within the organization.

### Learning from mistakes climate and employee work engagement

Work engagement is defined as “a positive, fulfilling, work-related state of mind that is characterized by vigor, dedication, and absorption” (Schaufeli et al., 2002, p. 74). Scholars have suggested that an optimal work environment is required for employees to be engaged. One meta-analysis found that organizational policies and practices are two important aspects that influence employee work engagement (Harter et al., 2002). In other words, these policies and practices create an expectation about employee behaviors that is perceived by all employees. Hence, organizational climate plays a role in employees’ work processes and work outcomes (Dickson et al., 2001).

The learning from mistake climate, as with the learning organizational climate, is closely linked to employee work engagement (Islam and Tariq, 2018). We build upon this suggestion with our understanding that the learning from mistake climate is similar to other forms of organizational climate. In addition, we argue that the learning from mistake climate provides a safe place in which employees can work that allows them to do their best and to remain communicative when mistakes are made (Weinzimmer and Esken, 2017).

### Learning from mistakes climate with the changing nature of work

The sudden emergence, and current continuation, of the COVID-19 pandemic in Malaysia in March 2019 has left many organizations unprepared. With many employees working from home to cope with reduced movement to prevent the spread of the coronavirus, the suggestion was that workplaces would never again be the same. van der Lippe and Lippényi (2020) state that working from home may be the norm in the future with physical workspace reduced, while the frequency of working from home will increase (Nikmah et al., 2020). Working in the workplace leads to employees taking on multiple roles at the same time. Consequently, working parents may make more mistakes, especially when working in a new working environment, that is, their homes. The sudden emergence of the pandemic has also called on us all to be prepared for the unexpected. The need for communication has become more important than before, with employees no longer meeting daily in their workplace (Carlson et al., 2013).

The pandemic has led to abrupt changes in the nature of work, with most employees having to work from home with no prior training to prepare them for this event. With these sudden changes, employees are faced with many new challenges. Lecturers, for example, need to equip themselves with technologies and teaching software to provide an optimal online learning experience for students (Pozo et al., 2021). In the current context, when employees, without prior training, are required to adapt to the pandemic and the changes at work, more mistakes are bound to happen. Hence, having a high learning from mistake climate denotes the important aspects of making those mistakes known, being communicative and having employees learn from one another, with this termed “social informal learning” (Crans et al., 2021). A high learning from mistake climate also acknowledges that mistakes are acceptable and can be used to improve the current system to reduce and prevent future incidents (Gu et al., 2013). When mistakes are embraced, employees will experience higher psychological safety climate and become more engaged at work, including working from home (Lemmetty, 2021).

## Materials and methods

### Development of the learning from mistakes climate scale

To develop the Learning from Mistakes Climate Scale (LMCS), a pool of items was developed that could be used to assess the learning from mistake climate, with these items derived from the relevant literature. Two expert panels (i.e., a researcher in organizational studies and a psychometric expert) was convened to select items that met the definition of the mistake learning climate in the workplace, specifically in Malaysia. Three experts within the industrial/ organizational field were asked a series of questions to verify on the clarity and wordiness, overlapping responses, content and appropriateness of the items (Simon, 2016). The experts found that all 23 items were direct, specific (relevant to workplace), and easy to understand. Face validity is supported. Finally, it is agreed with 23 items with a Likert scale ranging from “1” (strongly disagree) to “5” (strongly agree). Higher scores indicated a positive learning from mistake climate within an organization (see Appendix A).

### Participants

Ethics approval was obtained from Sunway University Research Ethics Committee (Ethics Approval No.: SUREC 2021/075). Data were collected from 554 working adults in Malaysia aged 18–66 years [mean age (standard deviation [SD]) =32.28 (12.40)], with 468 of these participants taking part in the retest study. The participants were recruited using convenience sampling *via* social media platform (e.g., Facebook, WhatsApp). There was no pressure from organizations for their employees to complete the scale. The inclusion criteria were that participants needed to be above 18 years old and were white-collar employees in a Malaysian-based company. Participants, working as interns, freelancers, or with working experience of less than 6 months were excluded (Table 1). All participants provided their informed consent and email address for retest purposes and answered the set of questions through a questionnaire developed in Google Forms. After 7–10 days, participants were asked *via* email to answer the Learning from Mistakes Climate Scale (LMCS) for retest purposes.

**Table 1:** Demographic information of participants (*N* = 554).

Demographic	*N* (%)/range	Mean (SD)
**Gender**
Male	239 (43.1)	
Female	214 (56.7)		Others	1 (0.2)	
**Ethnicity**
Malay	83 (15)	
Chinese	328 (59.2)	
Indian	80 (14.4)	
Others	63 (11.4)	
**Status**
Single	368 (66.4)	
Married	174 (31.4)	
Separated/divorced	8 (1.4)	
Widowed	4 (0.7)	
Working hours (per week)		42.89 (10.55)
Years working in current organization		6.27 (7.93)
**Occupation**
Professional	226 (40.79)	
Manager	140 (25.27)	
Service and sales worker	100 (18.05)	
Technical and associated professional	36 (6.49)	
Clerical support worker	35 (6.32)	
Others	17 (3.07)	
Size of organization (number of employees)	1–2,000,000	7,829 (94)

SD, standard deviation.

### Instruments

The learning climate scale (LCS), developed by Nikolova et al. (2014), was used to measure the organizational learning climate. The LCS consists of three subscales: Facilitation, Appreciation, and Error Avoidance, each with three items, totaling up to nine items. The LCS is administered using a five-point Likert scale ranging from “1” (not applicable at all) to “5” (fully applicable). Higher scores in Facilitation and Appreciation subscales, as well as a lower score in the Error Avoidance subscale, indicate better organizational facilitation of employees’ learning. Nikolova et al. (2014) provided evidence that the LCS has good convergent, divergent, and predictive validity. Moreover, the Cronbach’s alpha values ranged from 0.75 (error avoidance) to 0.89 (facilitation), indicating acceptable to good internal reliability.

Work engagement was measured using the Utrecht Work Engagement Scale 3-Item Version (UWES-3), developed by Schaufeli et al. (2006), which is an ultra-short version of the nine-item scale. The aim of UWES-3 is to assess work engagement using three items which represent the dimensions of vigor, dedication, and absorption. The UWES-3 is administered using a seven-point Likert scale of “0” (never) to “6” (always/every day). A higher score reflects higher work engagement. The UWES-3 is shown to have factorial validity as the scale can be discriminated from other assessment tools on job boredom, workaholism, and burnout. In addition, the scale yielded acceptable to good internal reliability, with Cronbach’s alpha values ranging from 0.77 to 0.85.

Psychological safety climate was measured using the Psychological Safety (PS) subscale from the Team Psychological Safety and Learning Behavior Survey (Edmondson, 1999). The Psychological Safety subscale is designed to assess team members’ shared beliefs of the psychological safety to take interpersonal risks, discuss failure, and speak openly in the team. The scale consists of seven items rated on a seven-point Likert scale from “very inaccurate” to “very accurate.” Items 1, 3, and 5 are reverse scored. A higher score indicates higher perception of team psychological safety. The Cronbach’s alpha value, reported by Edmondson (1999), was 0.82 demonstrating good internal reliability. Discriminant validity was also reported.

### Statistical analysis

All analyses were performed using IBM SPSS Statistics (SPSS), AMOS v.27 and MPlus 6. Confirmatory factor analysis (CFA) was then performed using maximum likelihood estimation and Robust maximum likelihood estimation (RML). Robust Maximum Likelihood (RML) method generates less biased standard errors and able to perform well facing different sample size and degrees of non-normality.

A model is considered to fit the data when the following values are obtained: chi-square/degrees of freedom (*χ^2^*)/df < 3.0, root mean square error of approximation (RMSEA) <0.08. An RMSEA value close to 0.05 or below suggests a good fit to the data, with a value up to 0.08 indicating a reasonable error of approximation (Browne and Cudeck, 1993; MacCallum et al., 1996; Steiger, 1998). The goodness of fit statistics Exhibits bias toward samples (GFI) > 0.90, Adjusted goodness of fit statistics (AGFI) > 0.80, comparative fit index (CFI) ≥ 0.90 (Bentler and Bonett, 1980; Bollen, 1989; Hu and Bentler, 1999), and standardized root mean square (SRMR) 0 ≤ SRMR ≤ 0.05. A SRMSR value between 0.00 and 0.05 indicates a good fit to the data and between 0.05 and 0.10 an acceptable fit (Hu and Bentler, 1999). Model modification will be conducted to explore the best-fitted model which fits the data more appropriately. Although empirical statistics are significant in modifying a model, the contents of the theory of empathy are of equal importance when making decisions to retain or remove an item (Bollen, 1989).

## Results

### Confirmatory factor analysis

Confirmatory factor analysis (CFA) was used to test the factor structure of the newly developed measure. A one-model was hypothesized and tested. A total of two nested models were explored to establish the most appropriate factor structure of LMCS (Table 2). In model 1, six items (item 6, 10, 16, 19, 20, and 21) were found to how relatively low standard factor loadings below 0.4. Modification index indicated to have covariance between e1 and e2, e3 and e4, e7 and e8, e11, and e12, and lastly e16 and e17.

**Table 2:** Fit indices of standardized maximum likelihood estimates (*N* = 554).

Model	*χ^2^*	df	RMSEA	GFI	AGFI	CFI	SRMR	*χ^2^*/df
1- factor model (23 items)	1003.007	230	0.080	0.838	0.806	0.826	0.064	4.361^**^
1- factor model (17 items)	425.353	113	0.071	0.911	0.880	0.926	0.0466	3.764^**^

** *p* < 0.001.

**Figure 1:**
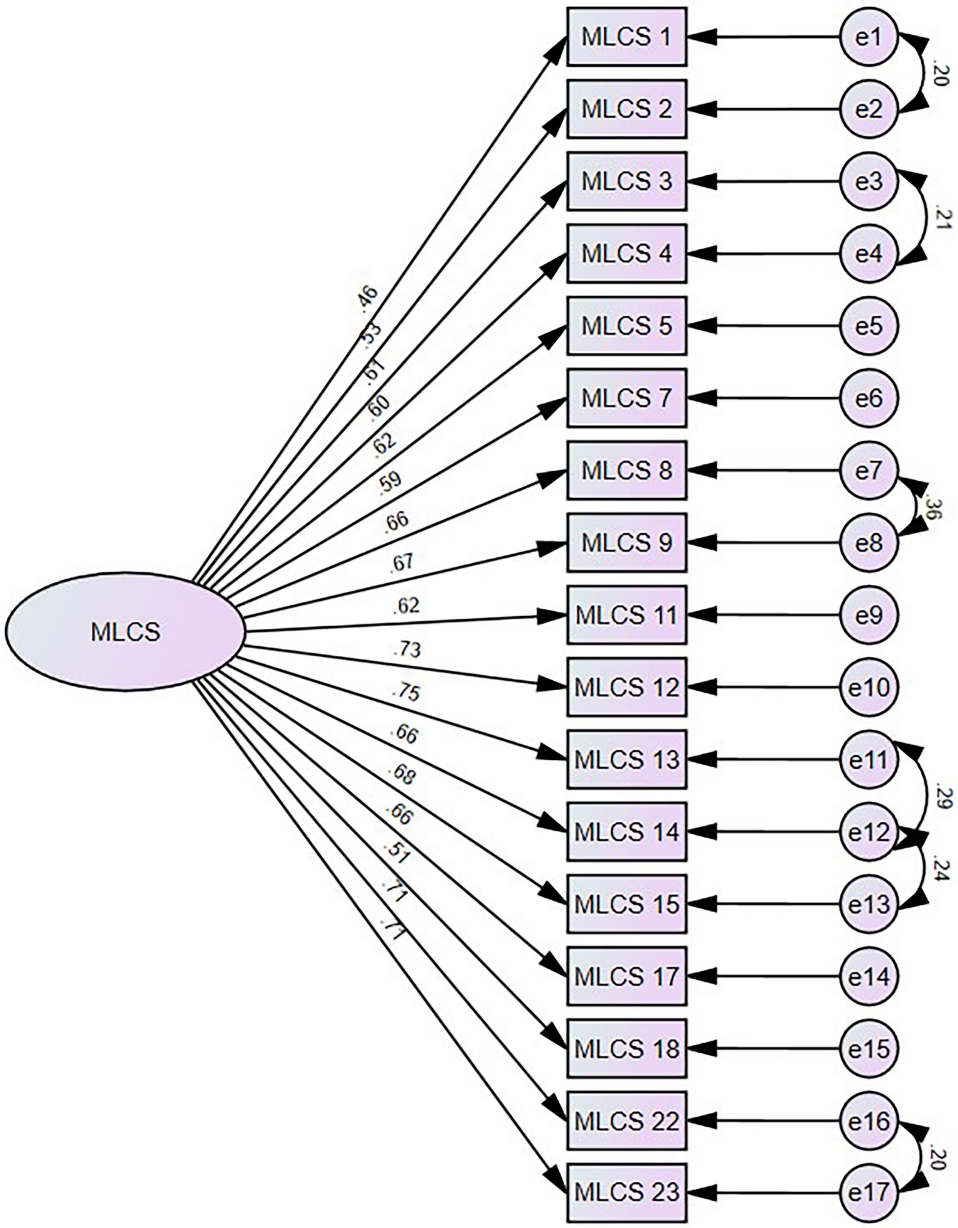
Factor structure of learning from mistakes climate scale using confirmatory factor analysis.

The final model fit analysis shows an adequate model of fit: *χ^2^*/[*df*] = 3.764, *p* < 0.001, CFI = 0.926, GFI = 0.911, AGFI = 0.880, RMSEA = 0.071, and SRMR = 0.0466. Standardized factor loadings for all items are moderately and highly correlated, with loadings ranging from 0.460–0.750 (Figure 1).

In addition, it is shown that the corrected chi-square value using robust ML is lower that the uncorrected ML value (425.353 vs. 338.188; Table 3). The large difference between the chi-square value also indicated the evidence of non-normality of the data. Therefore, model 2 is confirmed to be the finale fit of model of the LMCS.

**Table 3:** Comparison of model fit based on maximum likelihood (ML) and robust ML estimation in model 2.

Model fit statistics	ML estimation (without modification indices)	ML estimation (modification indices)	Robust ML estimation (without modification indices)	Robust ML estimation (modification indices)
Chi-square	626.450	425.353	434.990	338.188
DF	119	113	119	117
CFI	0.879	0.926	0.886	0.933
TLI	0.862	0.911	0.870	0.920
RMSEA	0.088	0.071	0.069	0.054
RMSEA 90% C.I	0.081; 0.095	0.064; 0.078	0.062; 0.076	0.0047; 0.062
Scaling correction factor for MLM	N/A	N/A	1.232	1.226

### The 17 items learning from mistake climate scale

In the current study, the mean (SD) for 17 items learning from mistake climate scale (LMCS) is 64.46 (11.16). A one-way ANOVA was performed to investigate if there are significant differences in LMCS, LCS, Psychological Safety subscale and UWES-3 among all different subgroups (e.g., Occupation, hours of working, gender, size of organization). We also looked at healthcare provider (*n* = 54) vs. other subgroups, we found that there were no significant differences among the scales. We also conducted correlations between LMCS, LCS, Psychological Safety subscale and UWES-3. All were positively significant except for Error Avoidance subscale that was negatively significant with others (see Appendix B).

### Convergent validity

Significant positive correlations are found between the Facilitating subscale (*r* = 0.562, *p* < 0.001) and the Appreciation subscale (*r* = 0.557, *p* < 0.001), with a significant negative correlation with the Error Avoidance subscale (*r* = −0.401, *p* < 0.001), from the LCS. These results reflect that the more that organizations provide resources that facilitate learning and show employees appreciation for desired behavior, the higher the learning from mistake climate at work, as well as the lower error avoidance in managing mistakes. Hence, convergent validity is supported.

### Criterion validity

A significant positive relationship is found between the Psychological Safety subscale and the LMCS (*r* = 0.578, *p* > 0.001). The result shows that when an employee perceives that he/she is psychologically safe in the workplace to bring up problems and to ask for help from team members, the workplace is more encouraging of employees learning from mistakes. Hence, criterion validity is supported.

### Concurrent validity

A significant positive relationship is found between the LMCS and the UWES-3 (*r* = 0.459, *p* > 0.001). The more a workplace encourages the culture of learning from mistakes, the more engaged are the employees: the more motivated they are to give more effort (vigor) and to feel more enthusiastic (dedication), and the more engrossed they are at work (absorption). Hence, concurrent validity is supported.

### Internal consistency

The Cronbach alpha coefficient for the 17 items LMCS is 0.921. Further analysis on reliability has been calculated with McDonald’s coefficient omega (ω; McDonald, 1999) without relying estimation of item factor loading and error variance in a CFA: McDonalds’s omega reliability = 0.922. This indicating excellent internal consistency.

### Test–retest reliability

Retest reliability was analyzed by calculating Pearson’s *r* coefficient values, following the assumption of normal distribution. All items showed significant correlation between coefficients (*r* = 0.196–0.398, *p* < 0.5). The total score from time 1 (test) and time 2 (retest) was showed significant correlation between coefficients (*r* = 0.611, *p* < 0.001). To control for potential systematic errors, intra-class correlations (ICCs) were calculated separately for single measures, with two-tailed tests conducted with an alpha level set at *p* < 0.05. The ICC values are found to be more robust to differences of absolute values between the two test sessions (test–retest; Weir, 2005). All items and total score show significant ICCs (intra-class correlations).

## Discussion

The study’s findings have shown that the developed Learning from Mistakes Climate Scale (LMCS) is validated and reliable. Its various types of validity are supported (i.e., convergent validity, criterion validity, and predictive validity). The scale also showed internal and test–retest reliability.

The LMCS is the first to address the mistake aspect of learning that occurs during daily tasks. Moreover, this scale is developed and validated within the Asian setting, that is, within a culture built on punishment and controls where employees are fearful of being reprimanded and, therefore, hide their mistakes (Zhang et al., 2008) which may cause worse consequences in the future (Khoreva and Wechtler, 2020). The developed scale not only acknowledges that mistakes can happen on a daily basis when carrying out work tasks, but that they may also serve as a source of knowledge for employees to brush up their skills on these tasks (Weinzimmer and Esken, 2017).

The importance of developing this scale is that the organization can tap into the level of mistake tolerance and utilize this as part of learning. This indirectly links to the level of communication and employee development within the organization (Weinzimmer and Esken, 2017). While Weinzimmer and Esken (2017) reported only that organizational learning was a mediator between mistake tolerance and work performance, the scale developed in the current study proposes that organizational learning should be derived from an organizational context, that is, the learning from mistake climate.

The developed scale is also different from previous scales as they are more focused on the learning climate within the formal training context. For example, the most popular climate for learning is the learning climate which touches the organizational level by emphasizing employee learning and the amount of training provided to employees (Eldor and Harpaz, 2016). The scale developed in the current study touches on the mistake aspect during the learning that occurs while carrying out daily tasks.

In a rapidly changing nature of work, organizational learning becomes nexus of organizational sustainability (Hermelingmeier and von Wirth, 2021). Hence, within the context of uncertainty, it is important for organizations to create a psychosocially safe space for employees to learn when mistakes happen. As organizations establish high psychosocial safety climate and learning climate, these serve as organizational resources that allow employee to be more engaged at work (Vieira dos Santos et al., 2021).

### Strength, limitations, and future directions

The current study has developed a facet-specific climate scale that allows researchers to tap into a specific area within an organization (Cooke and Rousseau, 1988). Our study’s findings complement the existing learning climate scales in the literature by developing and validating a Learning from Mistakes Climate Scale that focuses on mistake tolerance within the organization, allowing mistakes to be part of the employee learning process. With this scale, organizations can capture the collective climate of tolerating mistakes and treating them as part of the employee learning process. Human resources personnel may also use this scale to gauge the level of mistake tolerance within the organization and to create a more positive climate on this aspect (Bitsani, 2013).

While the LMCS does not measure the culture of the organization, it indirectly reflects that culture (Ehrhart et al., 2013). Carrim and Basson (2013) indicated that phenomena such as a learning climate within an organization resemble and reflect a learning culture. Several climate constructs can also be measured from a cultural perspective, thus providing a future research direction which links the existing organization culture, such as a hierarchical culture or a clan culture, to the learning from mistake climate and investigates how they are related.

In addition, while the LMCS taps into mistake tolerance by the organization and employees accepting mistakes as part of the learning process, the focus is more toward the organization and does not specifically address the social component of mistake learning (Amini and Mortazavi, 2012). In other words, the LMCS only looks at the overall perception of the organization toward mistake learning and less at the leader and colleagues with whom employees often interact. This other future research direction for the LMCS would investigate improvements to the understanding of the social aspect of mistake learning (Kandasamy et al., 2021).

Since this scale was developed in Malaysia, it cannot be generalized to other countries yet. A cross-cultural validation study should be conducted in the future to ensure its suitability in other countries (Güss, 2018). In addition, slightly more items were used in our study even though it only measured one aspect of the organizational climate (i.e., the learning from mistake climate). Following in the footsteps of the many scales that have developed a shorter version, a shorter version of this scale will be developed.

## Conclusion

A Learning from Mistakes Climate Scale (i.e., the LMCS) was developed and validated in this study. Full-time employees in Malaysia rated their experiences of making mistakes and learning at work, psychological safety, and work engagement. The scale was shown to have convergent, criterion, and predictive validity, as well as internal and test–retest reliability. The scale contributes to the literature on making mistakes at work and increases our understanding of how organizations can establish a positive learning climate by encouraging employees to be more transparent about their mistakes, while also emphasizing the need for leaders to be more mistake-tolerant. This climate will assist employees’ skill development; thus, it will help them to effectively carry out their tasks.

## Data availability statement

The raw data supporting the conclusions of this article will be made available by the authors, without undue reservation.

## Ethics statement

The studies involving human participants were reviewed and approved by Sunway University Ethics Board. The patients/participants provided their written informed consent to participate in this study.

## Author contributions

ML contributed to the initial idea. SW collected and analyzed the data. ML and SW did the write up for the paper. All authors contributed to the article and approved the submitted version.

## Funding

The study was funded by the Massey University School of Psychology REaDI Research Funding Scheme 2022.

## Conflict of interest

The authors declare that the research was conducted in the absence of any commercial or financial relationships that could be construed as a potential conflict of interest.

## Publisher’s note

All claims expressed in this article are solely those of the authors and do not necessarily represent those of their affiliated organizations, or those of the publisher, the editors and the reviewers. Any product that may be evaluated in this article, or claim that may be made by its manufacturer, is not guaranteed or endorsed by the publisher.
