# Supplementary material for: Learning from mistakes climate scale: Development and validation
Source: Front Psychol. 2022 Aug 8;13:911311. doi: 10.3389/fpsyg.2022.911311 (PMC9394742; doi:10.3389/fpsyg.2022.911311)
Supplement: Supplementary file 1 [file Table_1.pdf]

Appendix A

23-items of the Learning from Mistakes Climate Scale

No	Items	Strongly disagree	Disagree	Neutral	Agree	Strongly agree
1	When a mistake is made due to a lack of required resources for completing a task, employees provide an immediate solution.	1	2	3	4	5
2	When a mistake is made due to a lack of required resources for completing a task, employees are able to inform the management and the relevant department about the problem.	1	2	3	4	5
3	When an employee makes a mistake, his/her colleagues will talk to her, not for the purpose of blaming her, but rather as an opportunity for learning.	1	2	3	4	5
4	When employees make mistakes, they are not shy to share with others.	1	2	3	4	5
5	When employees make mistakes and if needed, they will inform their superior so the superior can inform other employees too as part of learning process.	1	2	3	4	5
6	When employees make mistakes, they do not hide those mistakes.	1	2	3	4	5
7	A question such as ‘‘why do we do things this way’’ is fully appreciated in this organisation.	1	2	3	4	5
8	This organization is open to suggestions we make in reducing future problems.	1	2	3	4	5
9	In this organisation, employees are encouraged to ask questions such as ‘‘is there a better way to produce the product or provide the service?’’	1	2	3	4	5
10	In this organisation, employees sometimes stop to reflect on the organisation’s work process.	1	2	3	4	5
11	Employees in this organisation often speak up to test assumptions about issues under discussion.	1	2	3	4	5
12	Employees in this organisation is supported so similar mistakes will not happen as improvement has been done.	1	2	3	4	5
13	Employees in this organisation feel ‘safe’ when they speak about their mistakes at work.	1	2	3	4	5

14	Employees in this organisation embrace mistakes as part of learning process.	1	2	3	4	5
15	Employees in this organisation learn from past mistakes of themselves and others.	1	2	3	4	5
16	Mistakes do not tarnish organisation's reputation and credibility.	1	2	3	4	5
17	Employees in this organisation is complimented when a good suggestion is given to improve a particular situation.	1	2	3	4	5
18	It is deemed alright to make mistakes at the workplace as part of learning process.	1	2	3	4	5
19	When mistakes happen, it is often not due to the employees' fault.	1	2	3	4	5
20	When mistakes happen, it is often not due to the system in place.	1	2	3	4	5
21	Mistakes of any form, small or large, is tolerated/ allowed.	1	2	3	4	5
22	Leader in this organisation hear us on the problems and mistakes we make at work.	1	2	3	4	5
23	My organisation welcomes any good suggestions to improve the system of doing work.	1	2	3	4	5
