# Supplementary material for: Learning from mistakes climate scale: Development and validation
Source: Front Psychol. 2022 Aug 8;13:911311. doi: 10.3389/fpsyg.2022.911311 (PMC9394742; doi:10.3389/fpsyg.2022.911311)
Supplement: Supplementary file 2 [file Table_2.pdf]

Appendix B

Correlation analysis for Learning from Mistake Climate Scale (LMCS), Learning Climate Scale (LCS), Psychological Safety subscale, and Utrecht Work Engagement Scale (UWES-3)

Scale/ subscales	1	2	3	4	5	6
1. LMCS	1					
2. Facilitation subscale	0.562**	1				
3. Appreciation subscale	0.557**	0.612**	1			
4. Error Avoidance subscale	-0.401**	-0.123**	-0.162**	1		
5 Psychological Safety subscale	0.578**	0.395**	0.428**	-0.472**	1	
6. UWES-3	0.459**	0.383**	.0396**	-0.143**	0.391**	1
